# Maternal Obesity Programming of Perivascular Adipose Tissue and Associated Immune Cells: An Understudied Area With Few Answers and Many Questions

**DOI:** 10.3389/fphys.2021.798987

**Published:** 2022-01-21

**Authors:** Adam Corken, Keshari M. Thakali

**Affiliations:** ^1^Arkansas Children’s Nutrition Center, Little Rock, AR, United States; ^2^Department of Pediatrics, University of Arkansas for Medical Sciences, Little Rock, AR, United States

**Keywords:** maternal programming, perivascular adipose, adipose inflammation, leukocyte, maternal obesity

## Abstract

At present, the worldwide prevalence of obesity has become alarmingly high with estimates foreshadowing a continued escalation in the future. Furthermore, there is growing evidence attributing an individual’s predisposition for developing obesity to maternal health during gestation. Currently, 60% of pregnancies in the US are to either overweight or obese mothers which in turn contributes to the persistent rise in obesity rates. While obesity itself is problematic, it conveys an increased risk for several diseases such as diabetes, inflammatory disorders, cancer and cardiovascular disease (CVD). Additionally, as we are learning more about the mechanisms underlying CVD, much attention has been brought to the role of perivascular adipose tissue (PVAT) in maintaining cardiovascular health. PVAT regulates vascular tone and for a significant number of individuals, obesity elicits PVAT disruption and dysregulation of vascular function. Obesity elicits changes in adipocyte and leukocyte populations within PVAT leading to an inflammatory state which promotes vasoconstriction thereby aiding the onset/progression of CVD. Our current understanding of obesity, PVAT and CVD has only been examined at the individual level without consideration for a maternal programming effect. It is unknown if maternal obesity affects the propensity for PVAT remodeling in the offspring, thereby enhancing the obesity/CVD link, and what role PVAT leukocytes play in this process. This perspective will focus on the maternal contribution of the interplay between obesity, PVAT disruption and CVD and will highlight the leukocyte/PVAT interaction as a novel target to stem the tide of the current obesity epidemic and its secondary health consequences.

## Introduction

Approximately 19% of all children and adolescents in the US can be categorized as obese (Sanyaolu et al., 2019). This is not merely a domestic issue as ∼370 million children worldwide are estimated to be either overweight or obese according to the World Health Organization (Di Cesare et al., 2019). These figures are expected to increase as the prevalence of childhood obesity has steadily increased with the recent reports indicating a 23 and 14% rise in developed and developing countries over the past decade alone. Increased weight gain during childhood has long-lasting consequences as overweight children have a 40–80% chance of being either overweight or obese as adults. The ramifications of obesity are numerous as it contributes to the development of several conditions such as type 2 diabetes, cardiovascular disease (CVD) and cancer, as well as a number of psychiatric disorders (Goldfield et al., 2010; Basen-Engquist and Chang, 2011; Park et al., 2014; O’Brien et al., 2016; Leitner et al., 2017; Chobot et al., 2018; Csige et al., 2018; Stone et al., 2018). For example, in epidemiological studies blood pressure and body mass index are positively associated across the lifespan, starting in childhood and early adolescence and continuing throughout adulthood (Stamler et al., 1978; Garrison et al., 1987; Jones et al., 1994; Rabkin et al., 1997; Chen and Wang, 2008; Forman et al., 2009; Ostchega et al., 2009; Suglia et al., 2013; Crump et al., 2016; Parker et al., 2016). Weight loss in obese patients leads to decreases in blood pressure and improved efficacy of anti-hypertensive drugs (Neter et al., 2003; Jordan et al., 2012). Importantly though not every obese individual will develop cardiovascular disease, obesity is an important contributor to cardiovascular disease risk at the population level. The consequences of obesity are wide-ranging and the severity of its burden continues to grow both in the US and abroad with children and adolescents now constituting a significant number of those afflicted.

There is no singular cause responsible for the development of childhood obesity. While initially believed to be solely attributed to a positive energy balance, studies have since elucidated that the origin and progression of obesity is multifaceted (Grundy, 1998; Chalk, 2004; Jukaku and Williams, 2021). In addition to energy balance, we now know that that other facets of nutrition (food refinement, meal timing, etc.) as well as environmental circumstances convey a compliance/resistance to an obesogenic state (Hruby and Hu, 2015; González-Muniesa et al., 2017; Xiao et al., 2019). Though many contributing factors are within an individual’s control, growing evidence indicates that maternal health at conception and throughout gestation affects offspring’s susceptibility to becoming overweight/obese (Smith et al., 2009; Heerwagen et al., 2010; Desai et al., 2013; Gaillard et al., 2014; Tie et al., 2014; Chang et al., 2019; Larqué et al., 2019). Indeed, maternal obesity predisposes offspring to being overweight or obese during adolescence and adulthood (Williams et al., 2014; Godfrey et al., 2017). As mentioned previously, the onset and progression of obesity elicits an increased risk to a number of maladies later in life, and there is growing evidence to indicate maternal obesity has an acute and long-lasting impact on cardiovascular health (Bridger, 2009; Umer et al., 2017; Wühl, 2019; Bashir et al., 2020).

In both humans and animal models, *in utero* exposure to maternal obesity is associated with increased risk of developing CVD later in life. Likewise, elevated maternal BMI during pregnancy correlates with early vascular disruptions in the offspring such as dyslipidemia, hypertension, myocardial fibrosis and ventricular hypertrophy (Huang et al., 2010; Fernandez-Twinn et al., 2012; Gambineri et al., 2020). Since approximately 60% of pregnancies in the US are born to either overweight or obese mothers it is imperative we elucidate the mechanistic purveyors of CVD progression in relation to maternal nutrition. Less clear, but likely important to cardiovascular outcomes associated with obesity, is the role of the perivascular adipose tissue (PVAT), which is the adipose depot that surrounds nearly all blood vessels. Over the years the function of PVAT has been increasingly linked to either the maintenance or decline of vascular health and function (Huang Cao et al., 2017). Its close proximity to the vasculature allows for it to act in a paracrine manner to regulate blood vessel pressure by eliciting the relaxation or constriction of the surrounding smooth muscle (Brown et al., 2014). Furthermore, as with other adipose tissues, PVAT is directly influenced by nutritional quality, with poor nutrition/weight gain leading to a loss of vascular relaxation (Qi et al., 2018). So though PVAT is influenced by individual nutrition, there are still many unknowns regarding the impact of the maternal environment during pregnancy on PVAT function. As both maternal nutrition and PVAT significantly impact an individual’s cardiovascular health, it is imperative to understand how they interact in an effort to not only better understand but also combat the growing number of obesity-related CVDs. As such we will outline the limited research investigating maternal programming’s impact of PVAT. Furthermore, as this topic is significantly underdeveloped, we will speculate the possible role of programming induced modulation of PVAT by supplementing knowledge gaps with tangential studies addressing the programming effect on alternate adipose depots and inflammation. As adipocyte changes and heightened inflammation are known drivers of PVAT disruption and vascular dysregulation, these observations will illuminate the possible interplay of programming/PVAT and serve as a launching point for future investigations.

## Perivascular Adipose Tissue—Composition and Function

PVAT is the adipose depot surrounding virtually every blood vessel in the body. Although its existence has been known for some time, it was often resected and disposed of prior to any vascular examination or experimentation. The widespread presumption of PVAT function was that it merely provided structural integrity to the adjacent vessel and was otherwise inconsequential. Since then, many studies have illustrated that PVAT is a significant regulator of vascular tone (Brown et al., 2014; Nosalski and Guzik, 2017; Cheng et al., 2018; Qi et al., 2018; Chang et al., 2020). This regulatory function is facilitated via the production of a milieu of soluble mediators (cytokines, chemokines, adipokines and small signaling molecules) that conveys the ability to govern vascular tension by directing the contractility of both vascular smooth muscle and endothelial cells of the vessel in a paracrine manner. The ability to generate such a diverse array of biologically active molecules is due to the heterogeneous composition of PVAT which includes not only adipocytes, but leukocytes, neurons and multipotent progenitor cells (Cheng et al., 2018). Traditionally adipocytes are categorized as either brown or white, which display either thermoregulatory or energy storage properties, respectively, while beige cells exhibit both brown and white characteristics. In our opinion, PVAT, however, represents a unique subset of adipose tissue as their principle function—modulation of vascular tone—deviates from that of classical brown, white and beige adipose depots. Interestingly, PVAT adipocyte phenotype varies depending on the vascular bed in which they reside. For example, in rodents, the adipocytes of PVAT covering the thoracic aorta have a predominantly brown adipose tissue phenotype, while those of abdominal aortic PVAT are composed of beige adipose tissue. Furthermore PVAT of the mesenteric artery and other arterial beds primarily contain white adipocytes (Qi et al., 2018). Less is known regarding PVAT composition in humans but current evidence indicates that PVAT adipocytes display both white and brown characteristics (Brown et al., 2014). Despite the outward appearance of the adipocytes within PVAT, the primary function of the tissue is the maintenance of vascular tension which is significantly different from the function displayed by brown and white adipocytes. This functional delineation could be attributed to the fact that PVAT adipocytes arise from SM22α^+^ progenitors, the same subset that yields vascular smooth muscle cells, while brown and white adipocytes are derived from Myf5^+^ and PDGFRα^+^ progenitors (Harms and Seale, 2013; Brown et al., 2014; Shin et al., 2020). The significance of SM22α in the development of PVAT adipocytes was confirmed via when the deletion of PPARγ using a Cre recombinase under the direction of the SM22α promoter resulted in an complete loss of PVAT development (Chang et al., 2012). Furthermore, lineage tracing of different sections from the thoracic aorta PVAT revealed SM22α^+^, Myf5^+^ and UCP1^+^ progenitors residing within the tissue (Ye et al., 2019). Though indispensably important to the overall function of the tissue, it is worth noting that adipocytes represent but one of many cell types comprising PVAT.

Many leukocyte populations including CD4^+^ and CD8^+^ T cells, B cells, natural killer (NK) cells, macrophages, mast cells, and neutrophils have been localized in PVAT. Indeed, PVAT depots have greater numbers of immune cells compared to canonical white and brown adipose depots (Kumar et al., 2020). PVAT phenotype and anatomical location affect leukocyte subpopulations, with mesenteric PVAT (white adipose phenotype) rich in CD68^+^ macrophages and thoracic PVAT (brown adipose phenotype) rich in T cells. Under basal conditions, macrophages of the M2 subtype are present within PVAT, which attenuates tissue inflammation via IL-10 production (Weisberg et al., 2003; Murray and Wynn, 2011). Likewise, both T and B lymphocytes reside within PVAT with regulatory T cell (Tregs), with B-1 cell subsets constituting the major cell phenotypes (Feuerer et al., 2009; Ait-Oufella et al., 2014). These cells aid in maintaining the health of the vasculature by exerting anti-atherogenic effects since Tregs produce anti-inflammatory cytokines and B-1 cells secrete IgM, which prevents foam cell formation. Additionally dendritic and natural killer cells as well as neutrophils and eosinophils are present but their function within PVAT is not as well characterized (Elgazar-Carmon et al., 2008; Wei et al., 2014; Wensveen et al., 2015; Saxton et al., 2020). There are also sex differences in immune cell subpopulations; PVAT from female rats have more NK cells and T cells compared to PVAT from males (Kumar et al., 2020). It is important to note that the cellular composition of PVAT is not fixed and is capable of reorganization as a result of disease, where modifications of the cell population dictate the effect PVAT will exert on the neighboring vasculature.

As mentioned previously the adipocytes and leukocytes within PVAT work in concert to maintain vascular tone by acting in a paracrine fashion, releasing vasoactive molecules that act on the underlying smooth muscle cells. Adipocytes secrete adiponectin which diminishes reactive oxygen species (ROS) production, suppress endothelial cell adhesion molecule expression and inhibits pro-inflammatory cytokine release (Ouedraogo et al., 2007; Gustafsson et al., 2013; Jenke et al., 2013). Adiponectin likewise stimulates endothelial cell production of nitric oxide (NO) which promotes vasodilation by inducing vascular smooth muscle relaxation (Sena et al., 2017). Adipocytes are also capable of generating NO and hydrogen sulfide (H_2_S) directly which also contributes to vasorelaxation (Victorio et al., 2016; Xia et al., 2016; Donovan et al., 2018; Cacanyiova et al., 2019). This delicate balance is easily susceptible to disruption via dietary changes such as increased consumption of refined carbohydrates and saturated fats, which can facilitate phenotypic changes in the adipocyte and leukocyte populations which has been observed in both human and rodent arteries (Nosalski and Guzik, 2017; Stieber et al., 2019).

## Perivascular Adipose Tissue—Disruption and Dysfunction

Studies from animal models demonstrate that improper nutrition leading to an obesogenic state also causes a “whitening” of thoracic PVAT wherein the characteristics of white adipocytes begin predominating (Chang et al., 2020). The whitening of PVAT results in a reduction in adiponectin and subsequent increased production of leptin which in turn drives down NO and H_2_S levels (Koh et al., 2008). The reduction in vascular relaxants coupled with leptin-induced increase in ROS production causes constriction of the adjacent blood vessel (Payne et al., 2014). Leptin likewise facilitates a transition toward an inflammatory state by increasing macrophage recruitment and driving the secretion of pro-inflammatory cytokines such as TNFα and IL-6 (Chen et al., 2010). Furthermore, dietary excess triggers a hypertrophic remodeling of adipose tissue wherein adipocytes rapidly expand in response to increase lipid intake (Jo et al., 2009; Henninger et al., 2014; Muir et al., 2016). The expansion of adipocyte size causes the cell to exceed the proper surface area-to-volume ratio thereby causing hypoxia due to limited O_2_ diffusion. This hypoxic state also contributes to the onset of an inflammatory state within PVAT.

In addition to contributing to adipocyte whitening, excess fat intake invokes changes in the leukocyte composition of PVAT (Almabrouk et al., 2018; Kumar et al., 2021). As indicated leptin dramatically enhances macrophage recruitment to PVAT so that they comprise 40–50% of all cells during obesogenic conditions whereas normally they represent less than 10% of the total population (Weisberg et al., 2003; Wynn et al., 2013). Moreover obesity elicits macrophage polarization toward the M1 class which produce the pro-inflammatory cytokines IL-6, IL-1β, and TNFα (Cancello et al., 2005; Kolak et al., 2007). Thus, the shift from the M2 to M1 phenotype coupled with the dramatic increase in macrophage number exacerbates the progression of an inflammatory state in PVAT. Additionally a high degree of T cell infiltration occurs alongside that of macrophages (Henrichot et al., 2005). After the onset of an inflammatory state, T_h_1, T_h_17, and cytotoxic T cells (CTLs) become the predominant classes of T cells present in PVAT which release of the potent pro-inflammatory cytokines IFNγ, TNFα, and IL-17. These and other soluble mediators augment the inflammatory status of PVAT which results in diminished NO and increased ROS levels thereby enhancing vasoconstriction and promoting insulin resistance in neighboring tissues (Wassmann et al., 2004; Nishimura et al., 2009; Zúñiga et al., 2010; Nguyen et al., 2013; Revelo et al., 2015). Though highly complex and entailing many cellular and molecular participants, PVAT dysregulation is largely the result of shifts in the resident adipose and immune populations. While great strides have been made to uncover the mechanisms behind this two-pronged process of tissue disruption regarding direct dietary changes, there is a paucity of data concerning the role maternal nutrition has on the phenotypic and functional modulation of adipocytes and leukocytes in PVAT. As we’ve discovered that maternal programming affects offspring health in other areas, it is possible that it may also influence PVAT adipocyte function or leukocyte composition in response to dietary stress. Previous reports highlighting a maternal factor contributing to the propensity for the onset of obesity in offspring demonstrates the potency of programming on adipocyte composition. Therefore, these examples illustrate a conceptual framework by which we can surmise the downstream effect of maternal nutrition on PVAT composition.

## Maternal Programming and Obesity

Excessive weight gain in women provides added health complications not shared by males as obesity hinders conception while also complicating gestation by increasing the likelihood of pre-eclampsia, gestational diabetes or a cardiovascular event (Shah et al., 2011; Moussa et al., 2016). Maternal obesity not only adversely affects the health of the mother but the offspring as well. Individuals born to overweight mothers have a high predisposition for becoming obese as several studies show a strong correlation between maternal obesity and the prevalence of obesity in both the childhood and adulthood of the offspring (Lukaszewski et al., 2013; Gaillard et al., 2014; Tie et al., 2014). Additionally, offspring born to obese mothers have an increased risk for coronary heart disease, stroke, diabetes, cognitive dysfunction and premature death (Reynolds et al., 2013; Godfrey et al., 2017; Cirulli et al., 2020). Interestingly, women who lost weight prior to becoming pregnant bore offspring that had reduced incidences of obesity throughout adolescence and early adulthood (Kral et al., 2006; Smith et al., 2009). These studies provide clear evidence of a programming effect in offspring driven by maternal health during conception and gestation. The long-term health impact of maternal programming, particularly with regards to obesity, is increasingly relevant since >60% of women in the US of reproductive age are overweight and ∼35% are obese (Flegal et al., 2012). As it stands, CVD is the number one cause of death in the US and a recent study of 37,000 individuals showed a heightened risk of developing CVD onset and early death in those born to obese mothers (Reynolds et al., 2013). Likewise a maternal high fat diet was associated with vascular hyper-responsiveness to contractile agonists, impaired endothelial function, and increased arterial blood pressure in rodents (Zaborska et al., 2016). So while maternal obesity increases the chances of offspring obesity, which itself contributes increased CVD risk, it appears that the maternal programming has a more direct influence on an individual’s cardiovascular health. Despite this demonstrable link between maternal obesity and offspring CVD risk, the specific mechanisms that mediate this programming remain unknown.

Obesity induces metabolic inflammation in adipose tissue, as well as other tissue systems (Xu, 2013; Wu and Ballantyne, 2020). Likewise, maternal obesity-induced metabolic inflammation during pregnancy and critical periods of fetal development can program inflammation in offspring tissues during fetal development and later on in life (Segovia et al., 2014; Chang et al., 2019). Rodent studies have shown that maternal high fat diet causes an increase in offspring pancreatic inflammation, as indicated by elevated TNFα levels, but does not have the same effect in hepatic tissues (Howie et al., 2013; Li et al., 2013). Similarly, elevated TNFα, IL-6, and IL-1β levels were observed in the hypothalamus of 90 day old rats born to mothers fed trans fats (Pimentel et al., 2012). Male mice exposed to maternal preconception and gestational high fat diet exhibited an increase in CD11c^–^ macrophages residing within subcutaneous and gWAT when fed a postnatal high fat diet (Chang et al., 2019). In a non-human primate model, maternal obesity was associated with altered transcription of genes related to antigen presentation, leukocyte transendothelial migration, and B cell receptor signaling pathways in fetal peripheral blood mononuclear cell (PBMC) (Farley et al., 2009). In humans, umbilical cord blood from babies born to mothers with obesity exhibited reduced esoinophils and CD4^+^ T cell counts, decreased monocyte and dendritic cell (DC) response to TLR ligands, and increased cord blood plasma IFNα_2_ and IL-6 (Wilson et al., 2015). However, there was no discernable difference in serum IL-6 or TNFα from human offspring at 12 or 57 years of age who born to obese mothers (van der Burg et al., 2016). Clearly, maternal obesity associated inflammation can developmentally program metabolic dysfunction in offspring through multiple physiological systems and mechanisms.

## Maternal Programming and Perivascular Adipose Tissue

It has been shown that maternal obesity is capable of eliciting an increased propensity for obesogenic adipogenesis (as opposed to developmental adipogenesis or organogenesis) in offspring (Desai and Ross, 2011; Lecoutre and Breton, 2015; Wu and Ballantyne, 2020) and there are several excellent reviews delineating the maternal programming effects on offspring adiposity (Lukaszewski et al., 2013; Lecoutre and Breton, 2015; Lecoutre et al., 2018). Though most of these conclusion have been drawn from observational studies, some investigations have begun to address the mechanistic cause behind this phenomenon. A recent rodent study demonstrated that a maternal high fat diet caused an increase in offspring adipocyte hypertrophy at 4 and 30 weeks post weaning relative to control diet offspring (Sellayah et al., 2019). However adipocyte counts did not vary indicating that adipocyte expansion was due to altered adipocyte hypertrophy and not modified adipogenesis. Additionally, the adipocyte progenitor genes *Fto* and *Zfp423* were significantly elevated in gonadal white adipose tissue (gWAT) in offspring born to mothers fed a high fat diet. These results corroborate an earlier report which observed a reduction in *Zfp423* promoter methylation in tissue from offspring born to high fat diet dams (Yang et al., 2013). Though insightful, these results were derived from investigating gWAT and general fetal tissue. It is unknown if maternal obesity has the same effect on PVAT as the limited number of investigations into maternal programming and PVAT focused primarily on changes in PVAT function instead of phenotypic adipocyte changes.

To date, only a few studies have attempted to address the effect of maternal programming on PVAT. A PubMed search with the terms “maternal programming” AND “perivascular adipose” or “maternal obesity” AND “perivascular adipose” yields only 8 publications. Maternal separation as a model of early life stress enhances the anticontractile effect of PVAT in male offspring fed a HFD, presumably through an enhancement of PVAT adiponectin expression (Loria et al., 2018). In regard to maternal obesity, PVAT from male offspring from HFD-fed Sprague Dawley dams exhibited a reduced anticontractile effect due to changes in NO bioavailability (Zaborska et al., 2016). Additionally, male offspring (on a high cholesterol diet) from apolipoprotein E deficient dams fed a HFD had an exaggerated inflammatory response in thoracic aorta PVAT with increased mRNA and protein expression of monocyte chemoattractant protein 1 (MCP-1) and TNFα, as well as increased number of residing MAC2^+^ and CD68^+^ cells compared to offspring from control-fed dams (Wakana et al., 2015). As PVAT houses leukocyte in addition to adipocytes which, depending on the circumstance, can accentuate or interfere with normal function, it is imperative to consider maternal programming’s influence on immunity and inflammation as well. Therefore, previous work establishing a maternal link to immune/inflammatory changes could provide a basis to intuit the possible consequences of programming on the phenotype of PVAT resident leukocytes.

It is worth noting that the majority of these studies did not investigate the changes in the composition or phenotype of leukocytes in a particular tissue and instead measured the levels of a few notable cytokines as a proxy for immune cell function. Furthermore, studies on maternal programming effects on adipose depots were chiefly concerned with changes to adipocytes. This course of investigations has led to a state where enough evidence has been generated to speculate on the adipocyte and leukocyte changes that may occur in PVAT in the presence of a maternal programming HFD insult. Importantly, the progenitor cells of PVAT differ from traditional white and brown adipose depots, suggesting that PVAT may signal and function differently from canonical adipose depots (Chang et al., 2012; Li et al., 2021). Since PVAT relies on both the adipocyte and leukocyte population to orchestrate the tissue’s regulation of vascular tone, it is critical that we build upon this prior knowledge with experiments designed to address the maternal programming effect on the phenotypic and functional changes of both the adipocytes and leukocytes residing within PVAT. Thus, studies to further our understanding of the relationship between the maternal *in utero* environment, obesity, inflammation, and PVAT constitute an important future endeavor.

## Discussion

For PVAT to maintain the necessary vascular tone to support cardiovascular health, it needs the proper adipocyte and leukocyte composition. While it is known that maternal health can contribute to other facets of the offspring’s health, little is known about its potential for predisposing an individual toward PVAT disruption either in the face of or absence of maternal malnutrition. Though there is some evidence to address maternal contributions toward adipogenesis, it is still unclear if this effect also occurs in PVAT. Moreover, we still do not know if maternal programming changes the basal phenotype of PVAT adipocytes, or what effect that might have on their secretory patterns and vascular regulation or what changes might occur when coupled with poor nutrition. The same questions can be presented for the resident leukocytes of PVAT. Is maternal programming capable of shifting either the number of immune cell subsets within PVAT or the phenotype and polarization of those cells? Is any potential programming present under normal circumstances or does it arise following the adoption of an improper diet by the offspring? By addressing these questions, we can begin to uncover the mechanisms by which maternal programming predisposes offspring to obesity and raises the risk of developing CVD.

## Author Contributions

AC and KT conceptualized the idea for the manuscript. AC performed the literature search and drafted the manuscript. KT revised and edited the final version of the manuscript. Both authors contributed to the article and approved the submitted version.

## Conflict of Interest

The authors declare that the research was conducted in the absence of any commercial or financial relationships that could be construed as a potential conflict of interest.

## Publisher’s Note

All claims expressed in this article are solely those of the authors and do not necessarily represent those of their affiliated organizations, or those of the publisher, the editors and the reviewers. Any product that may be evaluated in this article, or claim that may be made by its manufacturer, is not guaranteed or endorsed by the publisher.
